# Assessment of Transitioning from High-Potency to Low-Potency Inhibitors in Chronic Myeloid Leukemia (CML) Patients: The Downgrading-Impact (D-IMPACT) Project

**DOI:** 10.3390/cancers18101656

**Published:** 2026-05-20

**Authors:** Elisabetta Abruzzese, Monica Crugnola, Luca Garuffo, Uros Markovic, Malgorzata Monika Trawinska, Sara Barulli, Alessandro Maggi, Sara Galimberti, Daniele Cattaneo, Daniele Sannipoli, Mariella D’Adda, Elena Chiara, Massimiliano Bonifacio, Antonella Vita Russo Rossi, Germana Beltrami, Sabina Russo, Elena Crisà, Grazia Sanpaolo, Francesco Cavazzini, Giuseppina Loglisci, Carmen Fava, Valentina Giai, Anna Rita Scortechini, Barbara Scappini, Matteo Dalmazzo, Gianni Binotto, Monica Bocchia, Davide Facchinelli, Ambra Di Veroli, Sara Pasquina Pascale, Annapaola Leporace, Simona Bernardi

**Affiliations:** 1 Department of Hematology, S. Eugenio Hospital, Tor Vergata University, ASL Roma 2, 00144 Rome, Italy; malgorzatamonika.trawinska@aslroma2.it; 2 Hematology Unit and BMT, University Hospital of Parma, 43126 Parma, Italy; mcrugnola@ao.pr.it; 3 Bone Marrow Transplant Unit, Department of Clinical and Experimental Sciences, University of Brescia, ASST Spedali Civili di Brescia, 25123 Brescia, Italy; luca.garuffo@unibs.it (L.G.); simona.bernardi@unibs.it (S.B.); 4 Hematology and Bone Marrow Transplatation Unit, Azienda Ospedaliero Universitaria Policlinico G. Rodolico-San Marco, 95123 Catania, Italy; u.markovic@policlinico.unict.it; 5 Divisione di Ematologia di Muraglia, CTMO Ospedale San Salvatore, 61121 Pesaro, Italy; sara.barulli@sanita.marche.it; 6 Division of Hematology and Bone Marrow Transplant, Ospedale S.G. Moscati, 74010 Taranto, Italy; alexmag62@alice.it; 7 Department of Clinical and Experimental Medicine, Hematology, University of Pisa, 56124 Pisa, Italy; sara.galimberti@unipi.it; 8 Fondazione IRCCS Ca’ Granda Ospedale Maggiore Policlinico, 20122 Milano, Italy; daniele.cattaneo@unimi.it; 9 San Raffaele Scientific Institute—IRCCS, 20122 Milano, Italy; sannipoli.daniele@hsr.it; 10 S.C. Ematologia, ASST Spedali Civili di Brescia, 25123 Brescia, Italy; mariella.dadda@asst-spedalicivili.it; 11 IRCCS Fondazione Policlinico San Matteo, 27100 Pavia, Italy; c.elena@smatteo.pv.it; 12 Department of Engineering for Innovation Medicine, Section of Innovation Biomedicine, Hematology Area, University of Verona, 37134 Verona, Italy; massimiliano.bonifacio@univr.it; 13 Department of Precision and Regenerative Medicine and Ionian Area, “Aldo Moro” University School of Medicine, 70124 Bari, Italy; antonellavita.russorossi@policlinico.ba.it; 14 U.O. Ematologia e Terapie Cellulari, IRCCS Azienda Ospedaliera Universitaria San Martino, 16132 Genova, Italy; germana.beltrami@hsanmartino.it; 15 Hematology Section, AOU Policlinico “G. Martino”, University of Messina, 98124 Messina, Italy; sabina.russo@polime.it; 16 Candiolo Cancer Institute, FPO-IRCCS, 10060 Candiolo, Italy; elena.crisa@ircc.it; 17 UOC Ematologia e Centro Trapianti Cellule Staminali Emopoietiche, Fondazione IRCCS Casa Sollievo della Sofferenza San Giovanni Rotondo, 71013 San Giovanni Rotondo, Italy; g.sanpaolo@operapadrepio.it; 18 Hematology Unit, Arcispedale S. Anna, 44124 Ferrara, Italy; cvzfnc@unife.it; 19 UO Ematologia, Ospedale Vito Fazzi, 73100 Lecce, Italy; giuseppina.loglisci@asl.lecce.it; 20 Division of Haematology, AO Ordine Mauriziano di Torino, 10128 Turin, Italy; carmen.fava@unito.it; 21 Department of Clinical and Biological Sciences, University of Turin, 10124 Turin, Italy; 22 Division of Hematology, AUO Città della Salute e della Scienza, 10126 Turin, Italy; vgiai@cittadellasalute.to.it; 23 Clinica di Ematologia, Azienda Ospedaliera Universitaria delle Marche, 60126 Ancona, Italy; annarita.scortechini@ospedaliriuniti.marche.it; 24 Hematology Unit, Azienda Ospedaliero-Universitaria Careggi, 50134 Florence, Italy; scappini@aou-careggi.toscana.it; 25 Internal Medicine and Hematology, S. Luigi Hospital, University of Turin, 10043 Orbassano, Italy; matteo.dalmazzo@edu.unito.it; 26 Department of Medicine, Hematology and Clinical Immunology, University of Padua, 35128 Padua, Italy; gianni.binotto@unipd.it; 27 Hematology Unit, Azienda Ospedaliera Universitaria Senese, University of Siena, 53100 Siena, Italy; monica.bocchia@unisi.it; 28 Hematology Unit, Ospedale San Bortolo, 36100 Vicenza, Italy; davide.facchinelli@aulss8.veneto.it; 29 UOC Ematologia Ospedale S. Rosa, 01100 Viterbo, Italy; ambra.diveroli@asl.vt.it; 30 UOC Ematologia con Trapianto, Azienda Ospedaliera San Carlo, 85100 Potenza, Italy; sara.pascale@ospedalesancarlo.it; 31 Azienda Ospedaliera Sant’ Andrea Roma, 00189 Rome, Italy; aleporace@ospedalesantandrea.it

**Keywords:** CML, TKIs, dose, down-grading

## Abstract

**Background:** Since the advent of imatinib, more potent next-generation tyrosine kinase inhibitors (TKIs) and asciminib have expanded therapeutic options for chronic myeloid leukemia (CML). Treatment-free remission (TFR) is an important goal in CML management, but only ~30% of patients can achieve it, leaving many on lifelong therapy. TKIs are usually used in escalating order of potency, but in patients ineligible for or failing TFR, “downgrading” to safer, lower-potency agents may be advantageous. **Methods:** We analyzed this strategy in 157 patients across 29 Italian CML Campus centres. Data were collected via e-forms in July 2024. Prognostic scores at diagnosis did not influence downgrading. **Results:** The most downgraded TKIs were nilotinib (47%) and dasatinib (37%). Imatinib was the most frequent target agent (69.7%), followed by nilotinib and bosutinib. The majority underwent downgrading during first-line therapy and most after prior dose de-escalation. Adverse events were the leading reason for downgrading (79%). Notably, in 52.6% of cases, molecular responses improved afterwards. Twenty-one patients subsequently attempted TFR, with 17 (81%) remaining treatment-free at a median follow-up of 22 months. **Conclusions:** These results show that, beyond the known role of de-escalation, downgrading represents a new, feasible approach to maintaining long-term control, limiting toxicity and costs, and favouring TFR.

## Simple Summary

Patients with chronic myeloid leukemia often require long-term treatment with targeted therapies that effectively control the disease but may lead to cumulative side effects. In clinical practice, two main strategies are typically used to manage these issues: switching to a different drug, often a more potent one, or reducing the dose. In this study, we explore a third approach, termed “downgrading,” defined as a planned transition from a more potent drug to a less potent but better tolerated one. Using data from a large multicenter survey, we show that this strategy is frequently adopted in real-world practice, mainly to improve tolerability. Importantly, downgrading does not appear to compromise disease control and does not preclude the possibility of achieving treatment-free remission. These findings support downgrading as a practical and patient-centred strategy for long-term disease management and highlight the need for prospective studies to better define its role.

## 1. Introduction

Chronic myeloid leukemia (CML) is a clonal myeloproliferative disease characterized by a reciprocal translocation of chromosomes 9 and 22 [t(9;22)] [1]. It causes Philadelphia chromosome and the formation of new fusion gene (*BCR::ABL1*) encoding for the chimeric BCR::ABL1 tyrosine protein kinase [2]. BCR::ABL1 is targeted by tyrosine kinase inhibitors (TKIs) which have dramatically changed the fate of CML patients. Since the introduction of imatinib in 2000, other increasing-potency 2nd- and 3rd-generation TKIs have been added to the therapeutic pool of treatment possibilities for CML [3,4,5]. In particular, dasatinib, nilotinib, and bosutinib belong to 2nd-generation therapies, while ponatinib is a 3rd-generation treatment, mainly indicated for cases presenting with T315I BCR::ABL1 mutation associated with resistance to 1st- and 2nd-generation TKIs [6]. Recently, a new treatment, asciminib, was identified, which is an allosteric inhibitor that binds ABL1 and its myristoylated N-terminal in the myristoyl pocket, keeping the kinase autoinhibited [7,8,9]. Although the goal is to achieve treatment-free remission (TFR) for all patients, 70% of patients still require lifelong or otherwise long-term therapies [10,11,12]. This long period of treatment can create major problems, as TKIs are not free of side effects ranging from daily complaints that primarily impact quality of life (e.g., edema, cramps, diarrhea, skin reactions, headache, muscle pain) to adverse events of major clinical impact that may develop as a function of exposure time (e.g., renal failure, cardiovascular and/or pulmonary events) [13,14,15,16,17,18,19,20]. To manage often dose-dependent side effects/adverse events, a change in therapy (therapy switch) or modulation of dosage can be used [21,22,23,24,25,26,27]. TKI therapy is usually intensified in resistant cases; however, for patients on chronic treatment, stepping down to lower-potency, better-tolerated agents offers an alternative “downgrading” strategy whereby a high-potency drug is replaced with a lower-potency inhibitor with a “safer” profile [28,29,30]. For the purpose of this study, “downgrading” was defined as a planned transition from a higher-potency TKI to a lower-potency agent, primarily aimed at improving tolerability while maintaining disease control. This concept differs from “switching,” which refers more broadly to any change in TKI therapy regardless of direction or rationale [29].

Although no universally accepted classification of TKI potency exists, in clinical practice, second- and third-generation TKIs (e.g., dasatinib, nilotinib, ponatinib, asciminib) are generally considered higher-potency agents based on their ability to achieve faster and deeper molecular responses, whereas imatinib and, in some contexts, bosutinib are often perceived as lower-potency but better tolerated options. In addition, the introduction of both first- and second-generation generics alongside new therapies has increased the need to consider pharmaceutical costs in treatment decision both for healthcare systems and patients [31]. While the availability of generics does not directly impact clinical efficacy, it may influence treatment strategies by improving accessibility and supporting long-term sustainability of care [32]. In this context, transitioning from a more potent inhibitor to a less impactful and more cost-effective treatment may contribute to more efficient resource allocation, without compromising patient outcomes [33].

This is an important issue because patients who fail to obtain a TFR quickly go on to long-term therapy [6,34].

In the present study, we report the results of an Italian multicentric trial aimed at assessing for the first time the real-life application of TKI downgrading, the characteristics of patients undergoing this approach, the reasons driving treatment decisions and the associated clinical outcomes.

## 2. Materials and Methods

### 2.1. Trial Design

The project “Assessment of Transitioning from High-Potency to Low-Potency Inhibitors in Chronic Myeloid Leukemia (CML) Patients: The Downgrading-Impact (D-IMPACT) Project” was approved by the Italian CAMPUS-CML Working Group. According to applicable regulations and institutional policies, IRB approval and individual informed consent were not required as only retrospective, fully anonymized, aggregated data were collected [35,36].

Patient data were collected via an electronic case report form distributed to 58 Italian hematology CML Campus network centres from 24th to 31st of July 2024. The data were updated in October 2024. Surveys were returned by 29 centres. The survey collected information on age, sex, risk assessment, minimal residual disease (MRD) before and after TKI downgrading, type of downgraded TKI, reasons for downgrading, prior dose reductions, and TFR attempts. Prognostic risk was assessed using the SOKAL and EUTOS scores, which are validated tools commonly used in CML to stratify patients at diagnosis based on clinical and hematological parameters and to predict treatment outcomes [37,38,39].

There were four criteria for CML patients to be included in the survey. They had (i) to be on 2nd-generation or higher TKI therapy; (ii) to have switched from a higher- to a lower-potency inhibitor; (iii) to have documented side effects from high-potency TKIs; and (iv) to be 18 years or older.

Disease assessments, using the *BCR::ABL1*% International Scale molecular evaluation via real-time PCR, were performed when therapy was downgraded, at 3, 6, and 12 months afterwards, and at the final follow-up.

### 2.2. Aims

The primary aim of the study was to assess the real-world estimate of the extent of switching from higher to lower potency TKIs in Italian CML patients.

The secondary aims were to (i) identify the categories of patients involved in this therapeutic practice; (ii) analyze the reasons that led to downgrading of therapy; and, (iii) evaluate the clinical outcome of patients who switched to lower-potency drugs.

### 2.3. The Patient Cohort

One hundred and seventy CML patients, diagnosed with CML between 1996 and 2022 in 31 centres and who underwent the TKI downgrading procedure, were screened. As the study included only second-generation or higher TKIs, and focused on transitions from higher- to lower-potency agents, eighteen patients were excluded because they were treated with imatinib before downgrading, switched from dasatinib to asciminib, from nilotinib to dasatinib, or vice versa. A total of 152 enrolled patients were considered and analyzed.

### 2.4. Statistical Analysis

Descriptive statistical analyses were conducted to properly evaluate categorical variables including type of TKI, line of therapy, SOKAL and EUTOS risk, and MRD class. Data were analyzed using the software RStudio 2024.09.0 Build 375 to evaluate statistical significance through the Chi-squared and *t*-test analysis for contingency tables, Pearson’s correlation and chart generation.

## 3. Results

A summary of the main characteristics of the cohort of 152 CML patients is reported in Table 1.

Median age at diagnosis was 53 years (range 17–81 years), with 96 males (63.2%) and 56 females (36.8%). Treatment downgrading was attempted with a median time of 56 months from diagnosis (range 2–323 months) and 32 months from the start of the treatment with the downgraded TKI (range 2–162 months). We investigated the impact of the risk assessed via SOKAL and EUTOS scores, which were the routinely available prognostic tools within the dataset; the ELTS score, which has been shown to better predict CML-related mortality in the TKI era, was not uniformly available in this dataset and should be considered in future prospective analyses. SOKAL and EUTOS risk distributions are reported in Table 1. Among the survey participants, 56 (37%), 52 (34%), and 30 (20%) exhibited low, intermediate and high SOKAL risk, respectively. Data were not available for 14 patients (9%). In contrast, 79 (52%) and 14 (9%) survey participants had low and high EUTOS risk, respectively, with data not available for 59 patients (39%). Downgrading was applied across different baseline risk categories, suggesting that this strategy was not limited to patients with low-risk disease at diagnosis.

**Figure 1:**
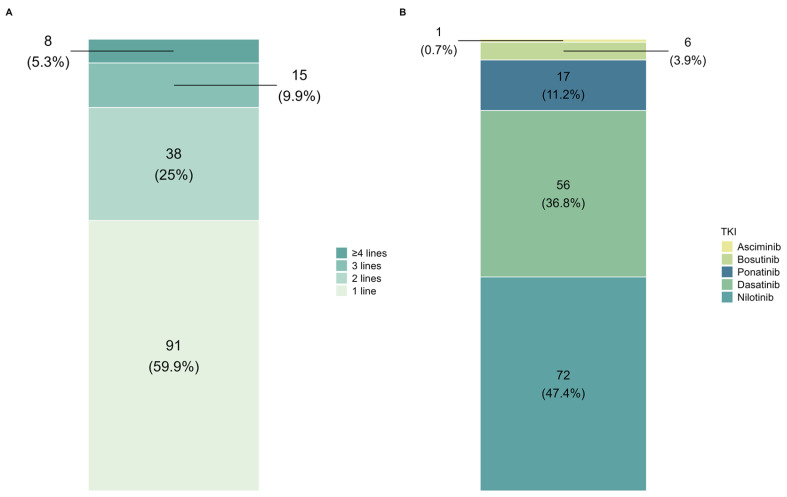
(**A**) Line of therapy immediately prior to downgrading. (**B**) Downgraded TKI.

The type of therapy prior to downgrading was analyzed to determine the impact and management strategy of downgrading. The majority of the patients (91, 60%) experienced downgrading after the first therapy line, followed by 38 patients (25%) and 15 patients (10%) who were downgraded after the second and the third line, respectively. Only eight (5%) patients experienced downgrading after at least four therapeutic lines (Figure 1A). The medications taken by patients before downgrading were nilotinib (*n* = 72, 47%), dasatinib (*n* = 56, 37%), ponatinib (*n* = 17, 11%), bosutinib (*n* = 6, 4%), and asciminib (*n* = 1, 1%; Figure 1B). The downgrading strategy was preceded by TKI dose de-escalation in 87 (57%) patients. Among them, 38/87 (43.6%) were receiving nilotinib, 33/87 (37.9%) dasatinib, 11/87 (12.6%) ponatinib, 4/87 (4.5%) bosutinib, and 1/87 (1.1%) asciminib. Dose de-escalation was performed mainly due to extra-hematological (*n* = 78, 90%) or hematological (*n* = 6, 7%) toxicity with the last TKI prescribed for a median of 32 months (range 3–162 months) before downgrading. Systematic dose data were not uniformly available across centres. Dose reduction for de-escalation was defined as any dose lower than the approved standard dose.

**Figure 2:**
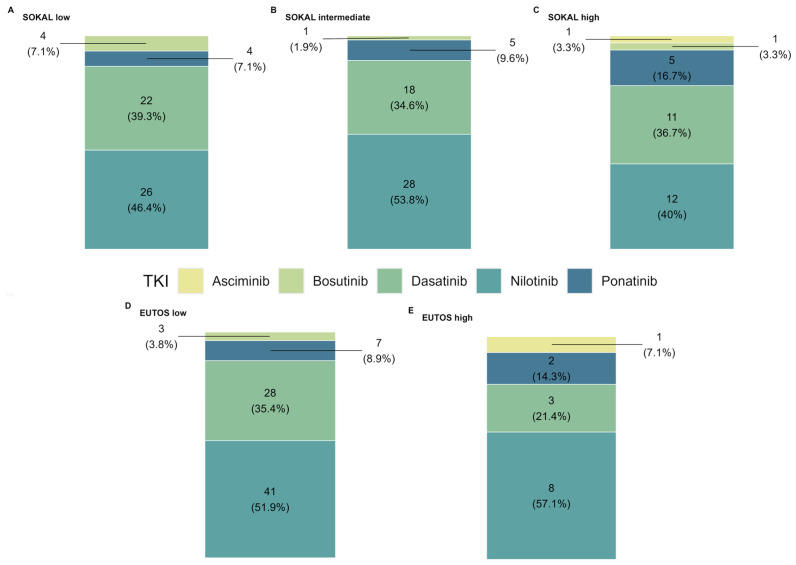
Downgraded TKI distribution according to SOKAL and EUTOS scores. (**A**–**C**) refer to SOKAL low, intermediate and high, respectively. (**D**,**E**) refer to EUTOS low and high, respectively. Statistical significance has been assessed using a Chi-squared test on a contingency table RxC, resulting in non-significant differences for both SOKAL and EUTOS scores.

Combining the SOKAL risk data with the type of therapy before downgrading, similar results were observed for each treatment in patients with low, intermediate, and high SOKAL scores (Figure 2A–C). The single patient receiving asciminib was reported descriptively and was not considered suitable for comparative interpretation. A comparison of EUTOS scores with treatment type prior to downgrading showed the same trend (Figure 2D,E). The pre-downgrading line of therapy was initiated at a median time of 1 month after diagnosis, according to the fact that the majority of patients experienced downgrading after the first therapy line.

**Figure 3:**
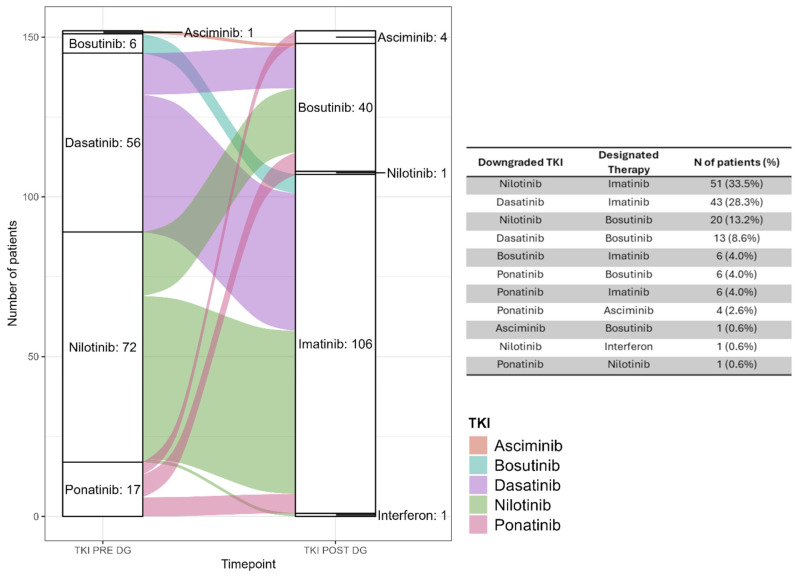
TKIs utilized before and after downgrading with downgrading practice recorded for all patients (152) reported in the table on the right.

To understand the merits of the downgrading strategy, the survey results revealed that 106 patients (69.7%) switched to imatinib, 40 (26.3%) to bosutinib, 4 (2.6%) to asciminib, 1 (0.7%) to nilotinib, and 1 (0.7%) to interferon alpha (Figure 3). The primary reasons provided for downgrading were the consequence of adverse events (120, 79%) and an elective choice to avoid/reduce adverse events (10, 6.6%). One patient was downgraded because of pregnancy, following the current guidelines [40], and was the only study participant who was switched to interferon alpha (Supplementary Figure S1). The most frequent switch was nilotinib to imatinib (51 patients, 32.5%), followed by dasatinib to imatinib (43, 27.4%). Complete switch practices are summarized in Figure 3.

We also analyzed the molecular response before and after downgrading, because downgrading unavoidably impacts the ability to achieve TFR. Post-downgrading molecular responses at 3, 6, 12 months (±7 days) and at the final follow-up have been analyzed. Data were available for 145/152 (95%), 140/152 (92%), and 148/152 (97%) patients at 3, 6 and 12 months, respectively. Results of patients with at least two timepoints have been considered.

**Figure 4:**
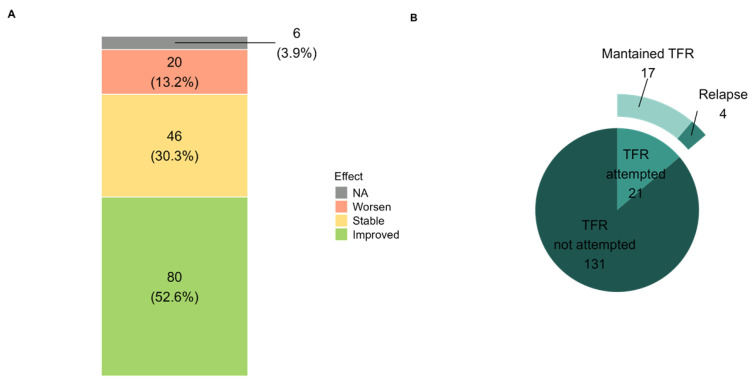
(**A**) Trend of molecular response after downgrading. (**B**) The scheme reports the TFR attempts and the capability of patients who experienced TKI downgrading to sustain the TFR.

For the purpose of this analysis, molecular response was classified as improved when BCR::ABL1 transcript levels decreased compared with the value recorded at downgrading, stable when the same molecular response category was maintained without clinically relevant increase, and worsened when an increase in *BCR::ABL1* transcript levels resulted in loss of molecular response category or required treatment reassessment. The analysis showed *BCR::ABL1* transcript levels lowering in 80 patients (52.6%), stable in 46 (30.3%), and worsening in 20 (13.2%) with no data for 6 patients (3.9%; Figure 4A). Genetic analysis of TKI-resistant mutations was performed in 3 out of 20 patients with worsened MR. Only one patient was carrying the TKI-resistant mutation *ABL1* p.T495S and switched to bosutinib. Among the two patients who resulted negative for resistance mutations, one started treatment with dasatinib and the other one with ponatinib because of loose of complete hematological response. Both achieved molecular response with this latter therapy line. Eight out of the 17 (47%) non-NGS-screened patients lost MR3.0. Among them, three experienced TKI dose adjustment because of the start of chemotherapy for solid tumours. In 9 out of 17 (53%) patients, we recorded the worsening of the molecular response, but they did not lose Major Molecular Response (MMR, BCR::ABL1 ≥ 0.1% on the International Scale, Supplementary Table S2) and remained under the less potent TKI. No correlation between the molecular response and the de-escalation strategy before downgrading the TKI was observed. To support this observation, molecular response outcomes were analyzed according to prior dose de-escalation, type of downgrading switch, and number of previous treatment lines. No statistically significant association was observed between molecular response and prior de-escalation, type of TKI switch, or number of previous lines. Given the limited number of events in some subgroups, this data should be considered exploratory.

Further analyses determined that the worsening of the molecular response post downgrading was not due to the TKI combination or by the number of pre-downgrading therapeutic lines.

An additional descriptive analysis was performed for the most frequent downgrading patterns, including dasatinib-to-imatinib, dasatinib-to-bosutinib, nilotinib-to-imatinib, and nilotinib-to-bosutinib. Molecular response outcomes after downgrading were broadly comparable across these groups, without evidence of a specific switch being associated with a higher risk of molecular worsening. These data are reported in Supplementary Table S1.

Among the entire cohort of 152 patients, 21 patients (14%) entered TFR, with 17 (81%) remaining free of treatment at a median follow-up of 37 months (range 3–104 months). All relapsed patients recovered the molecular response upon resumption of therapy (Figure 4B). During follow-up after downgrading, no case of disease progression to accelerated or blast phase was observed, and no CML-related deaths were reported in the analyzed cohort.

**Figure 5:**
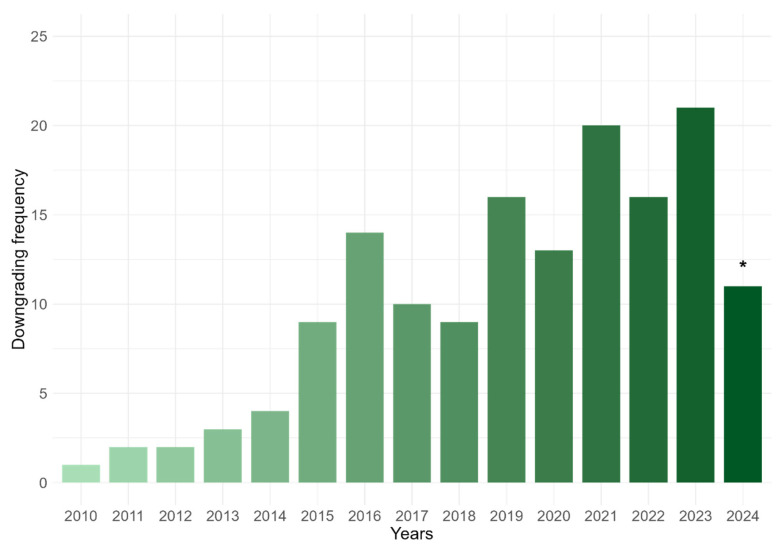
Report of the annual number of downgrading events recorded among patients included in the survey; * 2024 was limited to the end of June.

Finally, we evaluated the role of time in the practice of downgrading. Figure 5 reports the annual number of downgrading events recorded among patients included in the survey. Since the survey did not collect the denominator of all CML patients followed at each centre, these data should not be interpreted as the incidence or true frequency of downgrading in the overall CML population. The earliest downgrading occurred in March 2010, while the last one happened in May 2024. Surprisingly, switching to a less potent drug is a practice trending upward over the past 15 years (Figure 5).

Although adverse events were the leading reason for downgrading, the retrospective survey design did not include a systematic collection of adverse event grade, duration, or resolution after the switch. Therefore, a formal analysis of toxicity improvement after downgrading could not be performed. This limitation has been acknowledged in the Discussion and should be addressed in prospective studies.

## 4. Discussion

Even though the switch from a TkI to another is a frequent procedure as per clinical practice in CML patients [41], this exploratory survey shows for the first time that a downgrading approach can be safely considered in CML patients, particularly for long-term treatment. Based on the results reported here, switching to a less potent drug is regarded by physicians as a feasible and effective strategy, commonly chosen after first-line therapy in most cases [42]. Importantly, it is an approach applied across diverse patients with substantially different risk profiles. The majority of patients downgraded from nilotinib or dasatinib, reflecting the well-known long-term potential toxicity and tolerability challenges of second-generation TKIs. Indeed, the main reasons for downgrading were adverse events in 120 patients (79%) and the proactive choice to prevent or limit toxicity in 14 cases (9%).

As expected, imatinib and bosutinib were the most frequently chosen drugs for downgrading, likely due to their well-recognized safer profiles compared to other TKIs [43,44,45,46]. Importantly, physicians were deliberately given the freedom to assess and decide which drug they considered less potent. In clinical practice, bosutinib is generally perceived as less potent than other second-generation TKIs, even if results of the registrative trial are similar to the other 2nd-generation TKIs, probably due to the failure of the primary endpoint of the phase 3 BELA trial [47]. This trial failed to meet its primary efficacy endpoint (complete cytogenetic response (CcyR) rate at 12 months), due to early bosutinib discontinuations for adverse events. The subsequent phase 3 BFORE trial compared a reduced bosutinib starting dose of 400 mg QD with imatinib in adult patients with newly diagnosed CP-CML and showed that the MMR rate at 12 months (the primary endpoint) and cumulative CCyR rate at 12 months were both significantly higher in the boutinib arm, which determined approval of bosutinib in first-line settings [48,49,50].

The question of relative potency remains open for ponatinib and asciminib—both considered stronger than second-generation TKIs—although some physicians view asciminib as a more “downgraded” option compared to ponatinib [51,52].

In our case, four patients were switched from ponatinib to asciminib, with no cases reported in the opposite direction. Even though this is weak evidence, it could reflect the tendency to consider the preliminary apparently favourable balance of asciminib safety, compared to the risks of prolonged ponatinib use [53,54,55].

Although no head-to-head comparison can be made, a recent matching-adjusted indirect comparison (MAIC) study showed that ponatinib consistently achieved higher response rates than asciminib for both BCR::ABL1 IS ≤ 1% and MMR outcomes up to a year, particularly among those without a baseline response and also in the T315I subgroup [56].

Nowadays, CML treatment aims are focusing on survival, long-term safety, TFR, and sustainability [57,58,59]. This study’s data on the impact of downgrading on survival are reassuring. The result that no case of disease progression was observed after downgrading or during follow-up may indicate that stepping down to a less potent agent does not appear to compromise disease control when carefully selected and monitored. Maintaining long-term survival and durable remission remain the top priority in CML management, and the data presented here confirm the compatibility of downgrading with these objectives.

In terms of long-term safety, downgrading from a higher-potency TKI to a lower-potency one seems to be a valid strategy for minimizing treatment-related toxicity without sacrificing efficacy. Due to the retrospective design, a systematic and quantitative assessment of adverse event evolution after downgrading was not available and represents a limitation of the study. Although not formally captured in a structured manner, investigator-reported comments consistently indicated an improvement in treatment tolerability after downgrading across most centres, in line with the high rate of downgrading maintenance observed in this cohort. In this study, more than half of the patients (54.1%) even showed lower molecular responses while benefiting from a safer drug profile. This real-life experience has likely increased confidence among Italian hematologists in using downgrading as a tool to balance disease control with quality of life. Over the last decade, this practice has grown steadily as shown in Figure 5 and has emerged as a valuable alternative alongside other established strategies such as dose reduction, intermittent dosing, or treatment discontinuation.

Another key goal in CML today is TFR. Although the number of patients in this study was limited and generally did not meet standard TFR eligibility criteria, the results clearly show that downgrading does not preclude the possibility of achieving TFR. Therapy discontinuation was attempted in 21 out of 152 patients (13.8%), with a relatively low relapse rate of 23.8% (5/21 patients), supporting the notion that carefully selected patients may still reach TFR after downgrading. These findings highlight the need for larger studies to better define how downgrading can be integrated with TFR goals and support the evidence that the duration of response has more impact on TFR achievement than the type of TKI used. The introduction of new techniques, such as digital PCR, and the subsequent improvement in terms of MRD monitoring have already suggested this aspect [60,61,62,63].

Finally, sustainability is an increasingly important factor in CML management [64,65]. The upcoming patent expirations and the appearance on the market of second-generation generic TKIs will substantially reduce treatment costs, easing the burden on healthcare systems and broadening patient access.

This personalized approach of using less expensive drug or doses could also open opportunities to reallocate economic resources wisely, making it feasible to use newer, higher-cost drugs selectively and strategically, even in earlier lines of therapy [56,66].

## 5. Conclusions

Altogether, these results suggest that downgrading therapy aligns well with the main contemporary goals of CML management—safeguarding survival, optimizing safety, enabling TFR where appropriate, and supporting sustainable care.

These encouraging findings should inspire further prospective studies to better clarify how to incorporate downgrading into everyday clinical practice.

## Figures and Tables

**Table 1:** Demographic and Disease Characteristics of Patients at Baseline.

Characteristics	Number of Patients
Patients, *n*	152
M/F, *n* (%)	96/56 (63.2/36.8)
Median age at diagnosis, years (range)	53 (17–81)
SOKAL score, *n* (%)	
Low	56 (37)
Intermediate	52 (34)
High	30 (20)
Not available	14 (9)
EUTOS, *n* (%)	
Low	79 (52)
High	14 (9)
Not available	59 (39)
TKIs before downgrading	
Nilotinib	72 (47)
Dasatinib	56 (37)
Ponatinib	17 (11)
Bosutinb	6 (4)
Asciminib	1 (1)

## Data Availability

Raw data are available by corresponding author upon request.

## Supplementary Materials

The following supporting information can be downloaded at: https://www.mdpi.com/article/10.3390/cancers18101656/s1, Table S1. Molecular response outcomes according to downgrading pattern and prior treatment characteristics. Table S2. Definitions of molecular response levels in CML according to BCR::ABL1 transcript levels (International Scale, IS). Figure S1: Summary of downgrading causes.
