# Supplementary material for: Assessment of Transitioning from High-Potency to Low-Potency Inhibitors in Chronic Myeloid Leukemia (CML) Patients: The Downgrading-Impact (D-IMPACT) Project
Source: Cancers (Basel). 2026 May 20;18(10):1656. doi: 10.3390/cancers18101656 (PMC13204670; doi:10.3390/cancers18101656)
Supplement: Supplementary file 1 [file cancers-18-01656-s001.zip › cancers-4277068-supplementary.pdf]

Supplementary Materials

Supplementtray Table S1. Molecular response outcomes according to downgrading pattern and prior treatment characteristics. Statistical significance has been assessed via Chi-squared or Fisher's Exact test on RxC contingency table (ns = not significant).

	Molecular response after therapy				
Lines of therapy	Improved (%)	Stable (%)	Worsen (%)	NA (%)	p
1	45 (29.6)	26 (17.1)	16 (10.5)	4 (2.6)	ns
2	25 (16.4)	9 (5.9)	3 (2.0)	1 (0.7)	
3	7 (4.6)	6 (3.9)	1 (0.7)	1 (0.7)	
4	2 (1.3)	3 (2.0)	0 (0)	0 (0)	
5	0 (0)	2 (1.3)	0 (0)	0 (0)	
6 or more	1 (0.7)	0 (0)	0 (0)	0 (0)	
De-escalation pre-downgrading					
No	34 (22.4)	17 (11.2)	9 (5.9)	4 (2.6)	ns
Yes	46 (30.3)	29 (19.1)	11 (7.2)	2 (1.3)	
Downgrading combination					
Nilotinib → Imatinib	23 (15,1)	16 (10,5)	11 (7,2)	1 (0,7)	ns
Dasatinib → Imatinib	22 (14,5)	12 (7,9)	8 (5,3)	1 (0,7)	
Nilotinib → Bosutinib	12 (7,9)	6 (3,9)	0 (0)	2 (1,3)	
Dasatinib → Bosutinib	10 (6,6)	2 (1,3)	0 (0)	1 (0,7)	
Bosutinib →Imatinib	2 (1,3)	3 (2)	1 (0,7)	0 (0)	
Ponatinib → Bosutinib	4 (2,6)	2 (1,3)	0 (0)	0 (0)	
Ponatinib → Imatinib	4 (2,6)	1 (0,7)	0 (0)	1 (0,7)	
Ponatinib → Asciminib	2 (1,3)	2 (1,3)	0 (0)	0 (0)	
Asciminib → Bosutinib	0 (0)	1 (0,7)	0 (0)	0 (0)	
Nilotinib → Interferon	1 (0,7)	0 (0)	0 (0)	0 (0)	
Ponatinib → Nilotinib	0 (0)	1 (0,7)	0 (0)	0 (0)	

Supplementary Table S2. Definitions of molecular response levels in CML according to BCR::ABL1 transcript levels (International Scale, IS)

**Molecular Response Level**	**Definition (BCR::ABL1 IS, %)**	**Log Reduction from Standardized Baseline**
MR1	≤10%	1-log reduction
MR2	≤1%	2-log reduction
MR3 (MMR)	≤0.1%	3-log reduction
MR4	≤0.01%	4-log reduction
MR4.5	≤0.0032%	4.5-log reduction
MR5	≤0.001%	5-log reduction

Legend

Molecular response levels are defined according to BCR::ABL1 transcript levels measured on the International Scale (IS). Major molecular response (MMR), corresponding to MR3 (BCR::ABL1 ≤0.1% IS), is generally considered the optimal therapeutic milestone in routine clinical practice. Deeper molecular responses (MR4, MR4.5, MR5) reflect progressively lower levels of residual disease and are commonly used to assess eligibility for treatment-free remission (TFR).

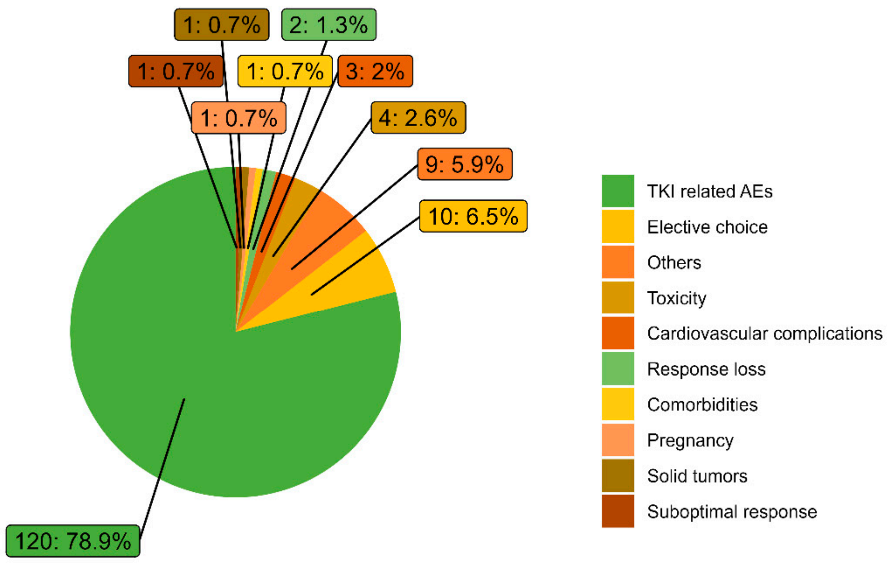

Supplementary Figure S1: Summary of downgrading causes.
